# Ursodeoxycholic Acid Exhibits Greater Inhibitory Effects on Cancerous HCT116 Colon Cells than on Noncancerous NCM460 Colon Cells

**DOI:** 10.3390/nu17061072

**Published:** 2025-03-19

**Authors:** Huawei Zeng, Bryan D. Safratowich, Wen-Hsing Cheng, Mary Briske-Anderson

**Affiliations:** 1 USDA-ARS Grand Forks Human Nutrition Research Center, Grand Forks, ND 58203, USA; bryan.safratowich@usda.gov (B.D.S.); mbanders71@gmail.com (M.B.-A.); 2 Department of Nutrition and Food Sciences, Texas Woman’s University, Denton, TX 76209, USA; wcheng1@twu.edu

**Keywords:** apoptosis, bile acid, cancer, cell cycle, pathway, proliferation

## Abstract

**Background/Objectives:** Ursodeoxycholic acid (UDCA), a hydrophilic bile acid, exhibits anti-inflammatory effects and attenuates the process of colon carcinogenesis. Certain healthy diets increase colonic UDCA concentrations, but its anticancer mechanistic actions remain largely unknown. We hypothesize that UDCA preferentially inhibits cancerous colon cell proliferation with a minimal effect on noncancerous colon cells. **Methods**: With human noncancerous NCM460 colon cell and cancerous HCT116 colon cell culture models, we performed biochemical, western blotting, PCR array, cell cycle, apoptosis, and immunofluorescent assays to determine the effects of UDCA treatment on colon cell proliferation and the underlying molecular mechanisms. **Results**: The inhibitory potential of UDCA against cell proliferation (via cell cycle arrest and apoptosis) was 90% greater in cancerous HCT116 cells than noncancerous NCM460 cells when treated with UDCA (0 to 0.4 mM) for 48 h. In UDCA-treated HCT116 cells, we identified 18 genes with ≥80% change (compared to untreated cells) in mRNA levels out of 93 apoptotic genes which were involved in caspase, death receptor, and NFκB pathways. At the molecular level, 0.4 mM UDCA reduced the protein level of the proto-oncogenic c-Myc gene but increased the putative tumor suppressor p21 gene (≥100%) via the ERK1/2/c-Myc/p21 pathway, which regulates cell cycle and apoptosis. These data are consistent with lower c-Myc but higher p21 expression in normal colon tissues compared to cancerous colon tissues. **Conclusions**: Collectively, UDCA inhibits cancerous HCT116 colon cells to a higher degree than in noncancerous NCM460 colon cells through cell cycle and apoptosis involving ERK1/2/c-Myc/p21 signaling.

## 1. Introduction

Colorectal cancer (CRC) is a serious health issue and a primary cause of mortality in the United States. About 50% of the population may have been diagnosed with a colorectal tumor by around the age of 70 years in the Western world [1,2]. Fortunately, about half of CRC cases can be prevented by adopting positive critical lifestyle habits such as a healthy diet and physical activity [3]. Tumorigenesis is a multistep process, and colon cancer is a chronic disease that can take 10 to 15 years to develop from a precancerous polyp into a full-blown cancer in which cell proliferation is out of control in the colon. While it is becoming increasingly evident that certain nutrients can prevent CRC [4], there are scant data examining the anticancer potential of dietary metabolites in the colon.

Fat consumption is associated with bile acid metabolism. In response to dietary fat, bile acids are synthesized from cholesterol in the liver. Subsequently, the gall bladder releases these bile acids to the small intestine, facilitating lipid absorption. In the colon, these primary bile acids (BAs) are deconjugated by bacterial bile salt hydrolase (BSH), and unconjugated bile acids are further modified to secondary bile acids [5]. Ursodeoxycholic acid (UDCA) is a secondary BA derived from the transformation of deoxycholic acid by colonic bacteria, plausibly improving intestinal barrier integrity and exhibiting anticancer properties in the colon [6]. While UDCA is commonly at the μM level in the colon [5,7], recent studies demonstrate that certain healthy diets (e.g., flaxseed meal) might greatly elevate the concentrations up to sub-mM levels [8,9,10,11,12]. Moreover, the results of dietary supplementation studies have suggested that there are medical benefits of UDCA, and the U.S. Food and Drug Administration has approved the use of UDCA to treat chronic cholestatic liver diseases such as primary biliary cirrhosis (PBC) [13]. UDCA concentrations at the mM level have been detected in serum, liver, intestinal, and adipose tissue samples in UDCA supplementation studies [6,13,14].

As an endogenous metabolite, UDCA is a major bile acid species found in bears (as used in traditional Chinese medicine) and is now also commercially synthesized. It is used to treat chronic liver diseases in humans as UDCA is the most hydrophilic and least toxic bile acid [6,15]. Prolonged UDCA administration not only reduces colonic cell proliferation but also decreases the risk of colorectal adenoma recurrence after removal in patients with PBC [16]. Similarly, animal studies also demonstrate that UDCA inhibits carcinogenesis and epithelium cell proliferation in the colon [17,18]. Although UDCA exhibits anti-inflammatory and chemoprevention properties, the underlying mechanism remains largely uncharacterized. An effective anticancer agent should exhibit a high efficacy in inhibiting cancer cell proliferation and carcinogenic pathways but a minimal effect on normal cells. At the cellular level, UDCA inhibits epithelial–mesenchymal transition, increases E-cadherin expression [19], and modulates the apoptotic process in epithelial cells in different tissues and physiological conditions [6,20,21]. To examine the impact of UDCA on colon cancer proliferation, many noncancerous and cancerous cell lines derived from different cancer stages are needed to generalize UDCA’s anticancer cell efficacy. However, there are few available cell lines of noncancerous colon cells; as an epithelial cell line, the NCM460 colon cell is derived from normal human colon mucosa without any genetic manipulation [22]. Among the several available cancerous colon cancer lines, the HCT116 cell line is derived from a colon epithelial carcinoma [23,24]. Thus, the NCM460 and HCT116 cells were chosen for the current report to provide a proof of concept for future studies.

At the base of colon crypts, undifferentiated crypt cells continuously divide and move toward the upper part of villi through cell cycle progression and cell proliferation within 48 to 96 h [25,26]. In this report, we concentrated on the impact of UDCA on colon cell proliferation, including cell cycle and apoptosis for up to 48 h, while examining their signaling molecules at the 24 h time point to reduce bystander effect.

## 2. Materials and Methods

### 2.1. Cell Cultures

The cell culture and cell proliferation methods were used as previously reported [27], with minor modifications. HCT116 colorectal carcinoma cells were purchased from American Type Culture Collection and we maintained these cells in DMEM (Invitrogen, Carlsbad, CA, USA) containing 10% fetal bovine serum (FBS; Sigma chemical Co., St. Louis, MO, USA). The nontransformed, noncancerous colon NCM460 cells were derived from human normal colon mucosa [22] and we initially maintained these cells in M3 Base medium (INCELL Corp., San Antonio, TX, USA) containing 10% FBS. Ursodeoxycholic acid (UDCA, w/purity > 99%) was purchased from Sigma Chemical Corporation (St. Louis, MO, USA). A 400 mM UDCA stock solution was prepared by dissolving UDCA in 100% ethanol. At ~80% confluency, the stock cells were passaged twice weekly and found to be mycoplasma free [28]. In all subsequent assays, both the HCT116 cells (at passages 22~40) and NCM460 cells (at passages 34~50) were grown in the DMEM medium with 10% FBS. Using a trypan blue exclusion method [29], we performed the cell proliferation assays with a Vi-CELL XR Cell Viability Analyzer (Beckman Coulter, Brea, CA, USA), and the cells were incubated in a humidified chamber at 37 °C with 5% CO_2_.

### 2.2. Cell Cycle and Apoptosis Analysis

The procedures for the cell cycle and apoptosis analysis were conducted as previously described [27], with minor modifications. In short, HCT116 cells were seeded at 300,000 and 160,000 cells/well in 6-well plates with UDCA treatment for 24 h and 48 h, respectively, while NCM460 cells were seeded with 550,000 and 280,000 cells/well in 6-well plates with UDCA treatment for 24 and 48 h, respectively. The cell cycle (DNA content) and apoptosis were analyzed using Guava Cell Cycle and Nexin^TM^ apoptosis kits (Cytek Biosciences, Fremont, CA, USA), respectively. For the cell cycle and apoptosis evaluation, each sample was analyzed with at least 5000 and 2000 single-cell events, respectively, using a Guava EasyCyte 6 HT flow cytometry analyzer (Cytek Biosciences, Fremont, CA, USA).

### 2.3. Western Blotting Analysis

Briefly, HCT116 cells in 6-well plates were treated with UDCA (0 to 0.4 mM) for 24 h before being lysed in an assay buffer for radioimmunoprecipitation (Cell Signaling Technology, Inc., Danvers, MA, USA). The subsequent cell lysates and western blotting assay were described in our previous reports [27,30]. Four independent experiments were conducted to collect cells. The following primary antibodies were used: c-Myc (Abcam, Cambridge, MA, USA), phosphorylated ERK1/2 (p-ERK1/2), ERK1/2, p21, PARP, and glyceraldehyde-3-phosphate dehydrogenase (GAPDH) as well as anti-mouse/rabbit HRP-conjugated secondary antibody (Cell Signaling Technology, Inc., Danvers, MA, USA). In Tris-buffered saline (TBS, 20 mM Tris, and 150 mM NaCl) buffer containing 5% non-fat dry milk, membranes were incubated with a primary antibody and then with a secondary antibody for 1 h at room temperature. Subsequently, blotted membranes were washed with TBS buffer (only) and incubated with chemiluminescence (ECL) solution. Finally, target proteins (including intensity of the immunoreactive bands) were detected and quantified by a LI-COR Odyssey Fc Imaging System (Lincoln, NE, USA).

### 2.4. Immunofluorescent Staining

As the cellular localization of transcription factor c-Myc directly affects its transcriptional activity, which controls cell proliferation [31], we then examined the impact of UDCA treatment on c-Myc localization. The method of immunofluorescent staining was used as described except for a few modifications [32]. Briefly, cells were seeded on microscope chamber slides (200,000 cells/chamber) in DMEM media containing 10% FBS in a 5% CO_2_ incubator at 37 °C overnight. The following day, the cells were treated with UDCA for 24 h. Images (~ 2000 cells per sample) were taken using a Nikon E400 microscope. Subsequently, the c-Myc protein content was quantified by Nikon NIS-Elements Version 5.02 software (Nikon Corporation, Melville, NY, USA).

### 2.5. Human Apoptosis PCR Array and Gene Enrichment Functional Analyses

Untreated and 48 h UDCA-treated cells were collected. Total RNA was isolated and treated with DNase using a RNase kit (Qiagen, Germantown, MD, USA). Concentrations and the integrity of the RNA samples were determined by NanoDrop spectrophotometry (Thermo Scientific, Waltham, MA, USA). The cDNA molecules were synthesized using 1 mg total RNA as the template by a reverse transcription reaction kit (Qiagen, Germantown, MD, USA). Subsequent cDNA samples were run on a TaqMan human apoptosis PCR array (Cat#4378701) (Thermo Scientific, Waltham, MA, USA) following the manufacturer’s protocol. For gene enrichment functional analysis, submitted genes were analyzed using the Kyoto Encyclopedia of Genes and Genomes (KEGG) and the Search Tool for the Retrieval of Interacting Genes/Proteins (STRING) database search engine [33].

### 2.6. Analysis p21 and c-Myc Gene Expression in Human Tumor Tissues

An online tool was used to examine gene expression in the human tumor tissues with the top five common cancer types in the U.S. [30]. We analyzed the p21 and c-Myc gene expressions in normal and cancer tissues of the breast, colon, lung, prostate, and skin using the TNMplot gene expression database (https://tnmplot.com/analysis/), accessed on 27 August 2024.

### 2.7. Statistical Analysis

Statistical analysis was performed as previously described [27], with minor modifications. Briefly, all the data from the UDCA-treated cells were analyzed by one-way analysis of variance (ANOVA) for comparing the mean values. The cell proliferation, cell cycle, and apoptosis were also analyzed by two-way ANOVA considering the cell type (HCT116 or NCM460) and concentration of UDCA and their interaction. We then used Tukey contrasts for post hoc comparisons. For c-Myc and p21 gene expression in human tissues (normal, n = 15,648 vs. cancer, n = 41,290), a Mann–Whitney U test (rather than the *t*-test) was used because of the large sample size. Using the comparative C_T_ method [34], we quantitated the real-time PCR array (HCT116 cell) data in which untreated cells were set as reference controls (fold change = 1). The subsequent fold change of the target gene data analysis was the same as previously described [27]. We used the relative expression ΔΔC_T_ values in the figure and table and reported them as a % change (2^−ΔΔCT^) in the text. All the data were presented as means ± SDs and analyzed by using JMP 18 software (SAS Institute, Inc, Cary, NC, USA).

## 3. Results

As UDCA is commonly at the μM to sub-mM levels in the colon of healthy human populations [6,7,8,9,10,11,12], 0–0.4 mM UDCA concentrations were chosen in our following experiments.

### 3.1. UDCA Exhibits Distinct Effects on Cell Proliferation Between HCT116 and NCM460 Cells

**Figure 1:**
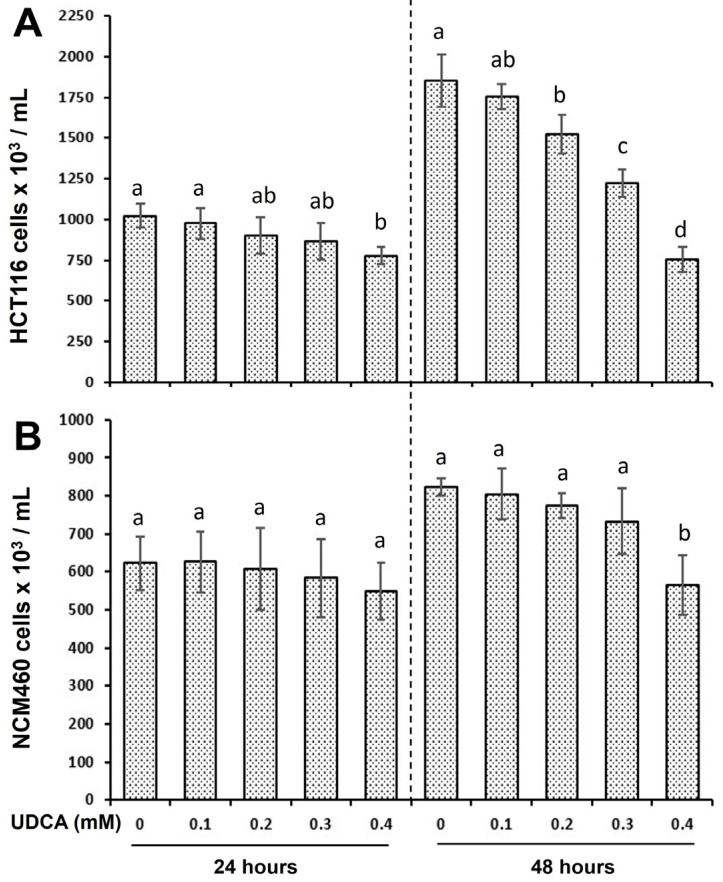
Distinct effects of UDCA treatment for 24 h and 48 h on the cell proliferation of (**A**) cancerous HCT116, and (**B**) noncancerous NCM460 colon cells. Values are means ± SD, n = 4 independent experiments. A significant interaction was seen between cell type (HCT116 vs. NCM460) and UDCA concentration at 48 h (*p* < 0.0001) by two-way ANOVA. At a given time point, when two values at a concentration in HCT116 or NCM460 cells share at least one letter, then the difference between them is not statistically significant. However, when two values do not have a letter in common, then the difference between them is statistically significant; *p* < 0.05.

The treatment with UDCA at 0.4 mM for 24 h inhibited cell proliferation by 24% in the HCT116 cells, but not in the NCM460 cells compared to the untreated cells (Figure 1A,B). Similarly, the treatment with UDCA at 0.2, 0.3, and 0.4 mM for 48 h inhibited cell proliferation by 18%, 34%, and 60% in the HCT116 cells, respectively, when compared to the untreated cells (Figure 1A,B). In contrast, only 31% proliferation inhibition in the NCM460 cells was detected when the cells were treated with 0.4 mM UDCA for 48 h. These differential effects on cell proliferation occurred because of the cell type (Figure 1).

### 3.2. Differential Effects of UDCA on Cell Cycle Progression and Apoptosis

**Figure 2:**
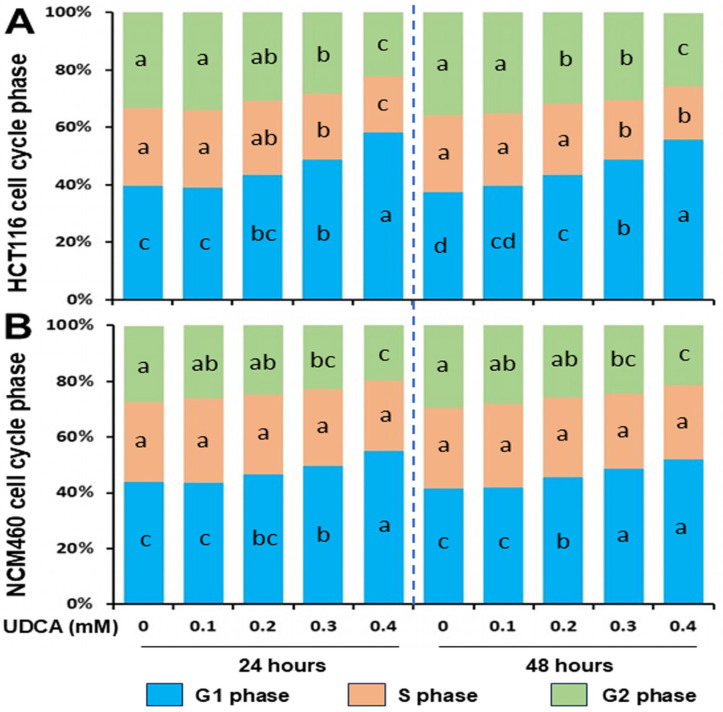
Distinct effects of UDCA treatment for 24 h and 48 h on the cell cycle progression of (**A**) cancerous HCT116, and (**B**) noncancerous NCM460 colon cells. Values of cell cycle phase (G1, S, and G2) are means, n = 4 independent experiments. A significant interaction was seen between cell type (HCT116 vs. NCM460) and UDCA concentration at 24 h (for G1-phase cells) and 48 h (for G1- and S-phase cells) by two-way ANOVA (*p* < 0.05). Within a cell line at a given time point, when two values of a specific cell cycle phase (same colored code) at a concentration share at least one letter, then the difference between them is not statistically significant. However, when two values do not have a letter in common, then the difference between them is statistically significant; *p* < 0.05.

As cell proliferation is regulated by cell cycle and apoptosis [35], we examined these two critical cellular events. Compared to untreated HCT116 cells, G1-phase cells were increased in a dose-dependent manner with a maximum of 47% at 24 h and 49% at 48 h under normal cell culture conditions (without synchronization); however, S-phase and G2-phase cells were decreased with a maximum of (29% at 24 h and 31% at 48 h) and (33% at 24 h and 29% at 48 h), respectively, when HCT116 cells were treated with 0.1, 0.2, 0.3, or 0.4 mM (Figure 2A, Table 1). Similarly, to a much lesser extent when compared to NCM460 cells, G1-phase cells were increased in a dose-dependent manner, including a maximum of 26% at 24 h and 25% at 48 h; however, G2-phase (but not S-phase) cells were decreased, with a maximum of 27% at 24 h and 27% at 48 h, respectively, when NCM460 cells were treated with 0.1, 0.2, 0.3, or 0.4 mM UDCA (Figure 2B, Table 1).

**Figure 3:**
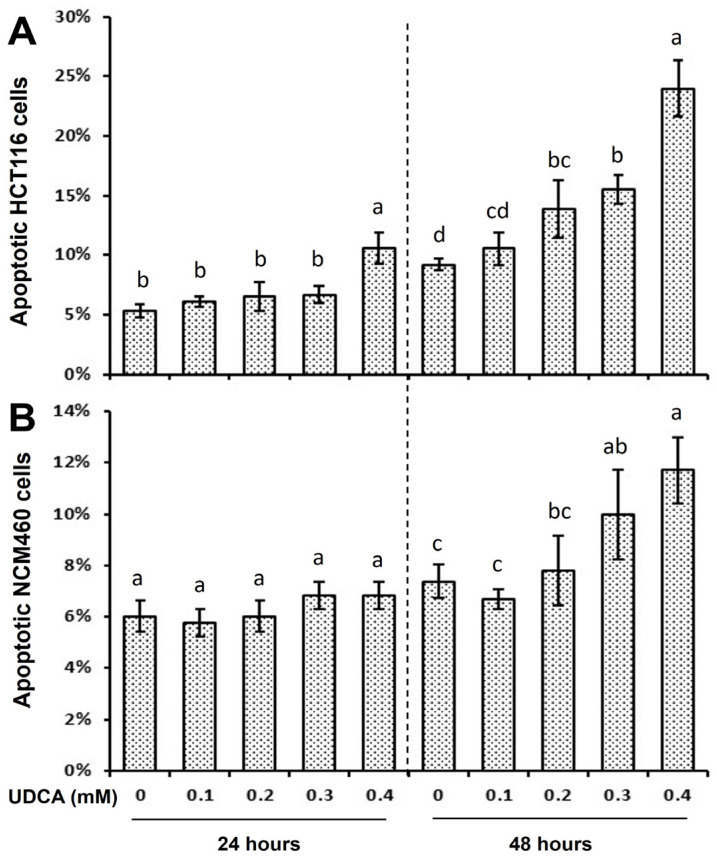
Distinct effects of UDCA treatment for 24 h and 48 h on apoptosis in (**A**) cancerous HCT116, and (**B**) noncancerous NCM460 colon cells. Apoptotic values are means ± SD, n = 4 independent experiments. A significant interaction was seen between cell type (HCT116 vs. NCM460) and UDCA concentration at 24 h and 48 h (*p* < 0.0001) by two-way ANOVA. At a given time point, when two values at a concentration in HCT116 or NCM460 cells share at least one letter, then the difference between them is not statistically significant. However, when two values do not have a letter in common, then the difference between them is statistically significant; *p* < 0.05.

Apoptotic cells were increased by 100% in the HCT116 but not in the NCM460 cells when treated with 0.4 mM UDCA for 24 h in comparison to the untreated cells (Figure 3A,B). Similarly, apoptotic cells were increased by 50%, 70%, and 160% at 48 h in the HCT116 cells when treated with 0.2, 0.3, and 0.4 mM UDCA, respectively, in comparison to the untreated cells (Figure 3A). Conversely, apoptotic cells were increased in a dose-dependent manner (but to a lesser extent) by 40% and 60% at 48 h in NCM460 cells treated with 0.3 and 0.4 mM UDCA, respectively, in comparison with the untreated cells (Figure 3B). The above distinct effect (between the two cell lines) of UDCA apoptotic potential was due to the cell type (Figure 3).

### 3.3. Effects of UDCA on Cell Signaling Molecules and c-Myc Cellular Localization

**Figure 4:**
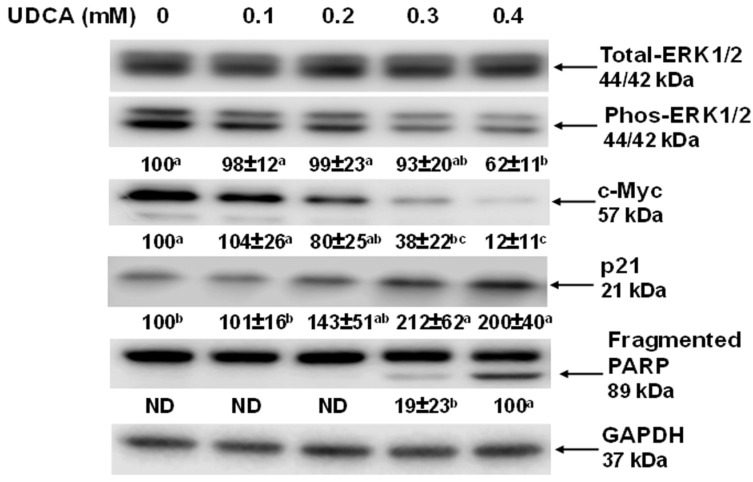
Effects of UDCA on signaling protein levels in HCT116 cells for 24 h. The signaling protein band intensity was normalized to GAPDH protein, and Phospho-ERK1/2 was further normalized to total ERK1/2 (Table S1). Representative western blotting images were displayed. Signaling protein values are means ± SDs, n = 4 independent experiments. For a given signaling protein, when two values share at least one letter, then the difference between them is not statistically significant. However, when two values do not have a letter in common, then the difference between them is statistically significant; *p* < 0.05.

As the UDCA exhibited a stronger potential for the inhibition of cell proliferation (via cell cycle arrest and apoptosis) in the HCT116 cells compared to the NCM460 cells, we only focused on UDCA-induced signaling molecules and apoptotic genes in HCT116 cells. Extracellular signal-regulated kinase ½ (ERK1/2) is pivotal in the cellular signaling pathway for survival signaling as a response to apoptotic stimuli when it is activated by phosphorylation [36]. Consistent with the effect of the UDCA on the HCT116 cell proliferation, the cell cycle progression and apoptosis (Figure 1, Figure 2 and Figure 3) phos-ERK1/2 level was decreased by 33% in HCT116 cells treated with 0.4 mM UDCA for 24 h (Figure 4), and the c-Myc protein levels were decreased by 66% and 90% in HCT116 cells treated with 0.3 and 0.4 mM UDCA, respectively (Figure 4). Conversely, the p21 protein levels were increased by 112% and 99% in HCT116 cells treated with 0.3 and 0.4 mM UDCA for 24 h, respectively (Figure 4). Similarly, fragmented PARP enzyme was detected in HCT116 cells treated with 0.4 mM UDCA for 24 h (Figure 4).

**Figure 5:**
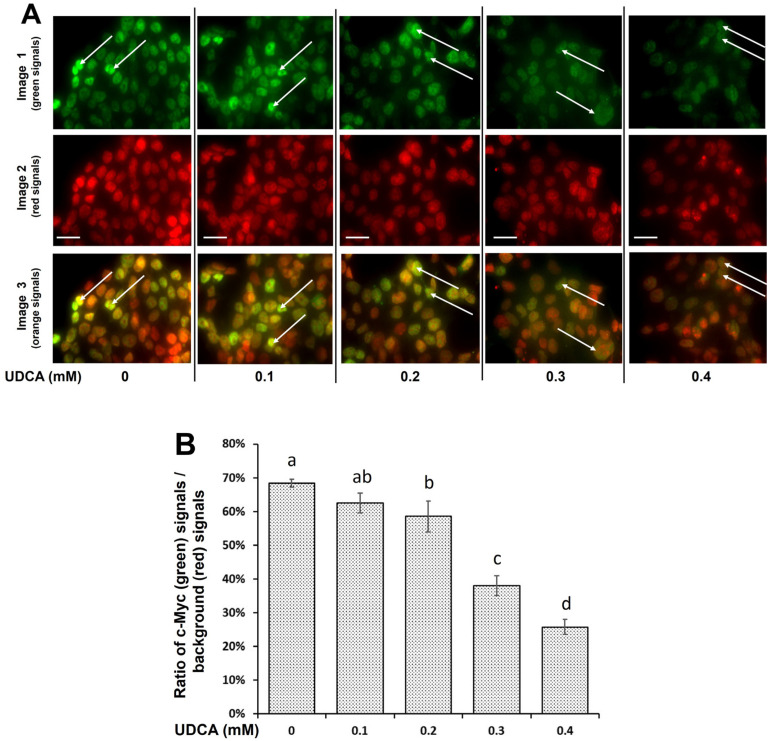
Effects of UDCA on c-Myc protein content and its cellular localization in HCT116 cells for 24 h. (**A**) Each sample is composed of three images at 1000× magnification. Image 1 (green signals): cells were labeled by anti-c-Myc antibody and subsequent anti-rabbit IgG (H + L), F(ab’)_2_ Fragment (Alexa Fluor^®^ 488 Conjugate). Image 2 (red signals): for reference of cell background, specifically nuclei, cells were mounted using fluoroshield with propidium iodide (PI) as a counterstain. Image 3 (orange signals): image 1 was superimposed on its respective image 2 to generate a composite image in HCT116 cells. White arrows indicate the concentrated c-Myc protein in the nuclei, and scale bar (25 μm) was embedded at the lower left portion of each image 2. (**B**) The percentage ratio of c-Myc protein (red) signal vs. cellular background (green) signals. Values are means ± SD, n = 4 independent experiments. The cellular fluorescent signals were measured by a fluorescent microscope coupled with Nikon NIS-Elements Version 5.02 software. When two values share at least one letter, then the difference between them is not statistically significant. However, when two values do not have a letter in common, then the difference between them is statistically significant; *p* < 0.05.

The c-Myc subcellular localization and its protein content play a critical role in regulating cell proliferation [31]. The c-Myc protein in the nucleus at 24 h was decreased by 41%, 62%, and 74% in HCT116 cells treated with 0.2, 0.3, and 0.4 mM UDCA, respectively, when compared to the untreated cells. In addition, more c-Myc protein (green signals) was located around the nuclei in the untreated cells (0 mM UDCA) compared to the HCT116 cells treated with 0.3 and 0.4 mM UDCA, respectively (Figure 5).

### 3.4. Differential Effects of UDCA on Apoptotic Gene Expression

**Figure 6:**
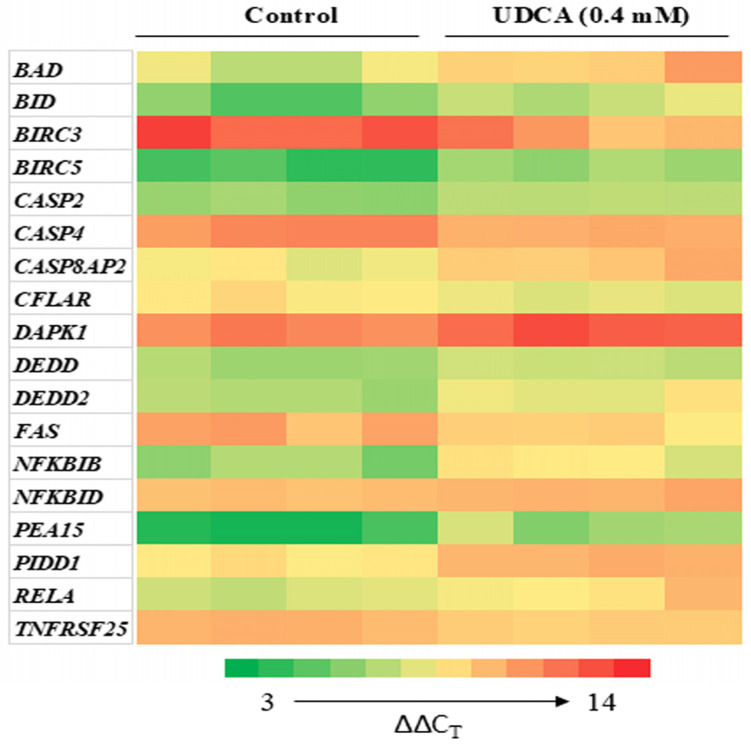
A heat map of 18 altered (either increased or decreased) genes which were annotated with gene ID when HCT116 cells were treated with UDCA (0.4 mM) relative to untreated cells for 48 h. The ΔΔC_T_ values are color-coded and each column represents a set of data from one independent experiment, n = 4 (the lower the gene expression, the higher the ΔΔC_T_ values). Means of all 18 genes’ ΔΔC_T_ values differ; *p* < 0.05 after FDR-corrected Student *t*-tests.

**Figure 7:**
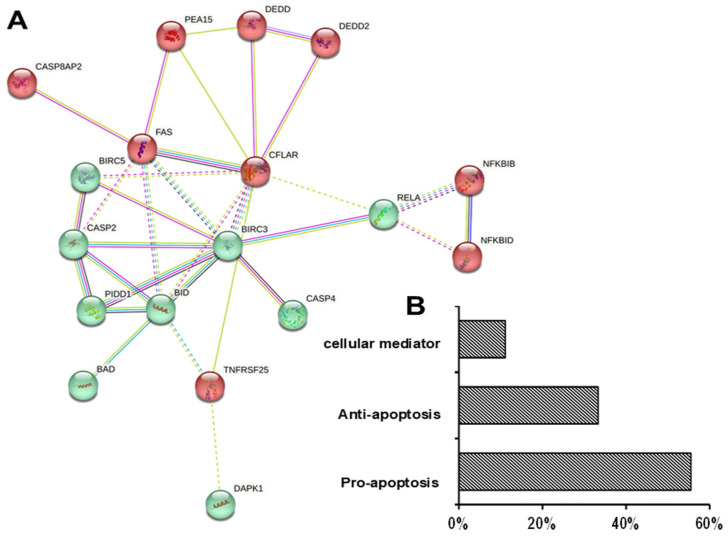
Cluster analysis of 18 differentially expressed genes in HCT116 cells treated with (0 or 0.4 mM) UDCA for 48 h. (**A**) There were 2 major protein–protein interaction clusters (red and green filled circles) in these 18 genes: in the figure, a solid line stands for a strong interaction and a dashed line stands for a weak interaction. However, both solid lines and dashed lines are color-coded as follows: (**--**) curated databases; (**--**) co-expression; (**--**) experimentally determined; (**--**) gene neighborhood; (**--**) text mining; (**--**) protein homology. (**B**) The overall percentage of cellular mediator, anti-apoptotic, or pro-apoptotic genes in these 18 genes.

Subsequently, we examined the critical genes involved in UDCA-induced cell apoptosis. At 48 h, the 0.4 mM UDCA-treated HCT116 cell sample exhibited the highest percentage of apoptotic cells among all the samples (Figure 3). Therefore, the 0.4 mM UDCA-treated HCT116 cell sample at 48 h was chosen for analyzing apoptotic gene expression. Using a human apoptosis PCR array to examine 93 key apoptosis-related genes, we identified 18 differentially expressed genes. Except for an increase (≥65%) in the mRNA levels of *BIRC3*, *CASP4*, *CFLAR*, *FAS*, and *TNFRSF25*, the mRNA levels of *BAD*, *BID*, *BIRC5*, *CASP2*, *CASP4*, *CASP8AP2*, *DAPK1*, *DEDD*, *DEDD2*, *NFKBIB*, *TA-NFKBH/NFKBID*, *PEA15, LRDD/PIDD1*, and *RELA* were decreased by at least 23% compared to the untreated cells (Figure 6, Table 2). When expressed, these 18 genes are primary signaling molecules in the BCL-2, Fas/TNF, death receptor, IAP protein, and caspase/NFκB pathways through protein–protein interactions. Proteins in the TNF, NFκB, and death receptor pathways were mainly in one cluster that interacted with the other cluster in the BCL-2, p53 and IAP pathways, although caspase genes were identified in both clusters (Figure 7). Further analysis indicated that 2, 6, and 10 genes contributed to cellular mediators, anti-apoptosis, and pro-apoptosis, respectively (Figure 7, Table 2).

### 3.5. c-Myc and p21 Expression in Human Normal and Colon Cancer Tissues

**Figure 8:**
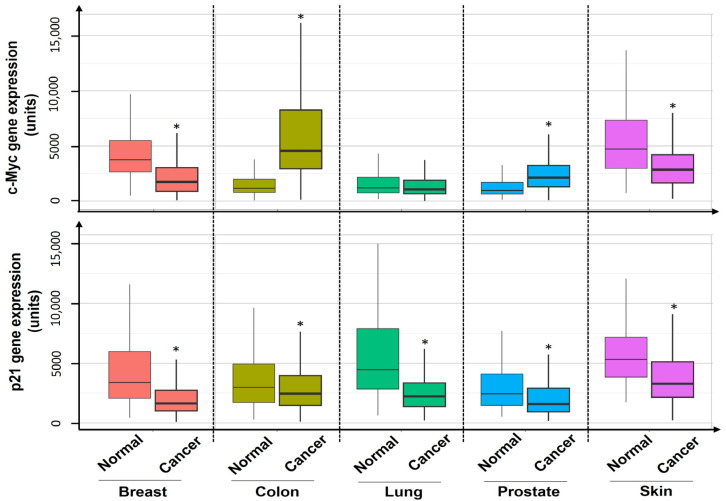
c-Myc and p21 gene expression in five common cancer types. Breast, colon, lung (sc), prostate, and skin tissues (normal, *n* = 15,648 vs. cancer, *n* = 41,290). Means of c-Myc and p21 gene expression (mRNA values) differ; * *p* < 0.05.

To gain clinical insights into the expression of c-Myc and p21, we analyzed their gene expression levels in 15,648 normal vs. 41,290 cancer tissues utilizing the TNMplot gene expression database (https://tnmplot.com/analysis/, accessed on 27 August 2024). The c-Myc mRNA levels of colon and prostate cancer types were increased by at least 100% relative to their respective normal tissues; by contrast, the c-Myc mRNA levels of breast and skin types were decreased by at least 60% in comparison to their respective normal tissues (Figure 8). In contrast, the p21 mRNA levels of all the above (five) cancer types were decreased by at least 25% compared to their respective normal tissues (Figure 8).

## 4. Discussion

The biological function and underlying mechanism of UDCA actions are multifactorial. UDCA is the least toxic and most hydrophilic bile acid among the colonic bile acids, and its chemo-preventive potential has been proposed [6,19]. We hypothesized that UDCA plays differential roles in the proliferation of cancerous and noncancerous cells via cell cycle and apoptosis modulation. In the present study, although we did not determine the cell number at 0 h (Figure 1), the percentage of cell confluency (observed when checking cells daily under a microscope) increased during the experimental 48 h. With respect to cell proliferation, UDCA was much more effective in inhibiting cancerous HCT116 cells than in noncancerous NCM460 cells (Figure 1). Similarly, there were increased G1-phase and decreased S- and G2-phase cell populations in HCT116 cells treated with UDCA when compared to untreated cells, and the extent was greatly reduced in NCM460 cells (Figure 2, Table 1). Along the same lines, UDCA was more effective in the induction of apoptosis in HCT116 cells than in NCM460 cells (Figure 3). These data indicate that the stronger potential of cell cycle arrest and apoptosis may be the mechanistic basis for the increased inhibitory efficacy of UDCA on cancerous HCT116 colon cells compared to noncancerous NCM460 colon cells. This observation is consistent with the ideal that an effective anticarcinogenic agent should preferentially target cancer cell proliferation with a limited impact on normal cells [37]. Therefore, we subsequently focused on the molecular mechanism of UDCA actions in HCT116 colon cancer cells.

The ERK1/2 pathway is a major cellular signaling pathway that regulates cell proliferation, cell cycle, apoptosis, differentiation, and cell survival [38,39]. In a variety of cancers, ERK1/2 signaling is de-regulated because of loss-of-function mutations in core components and the subsequent development of inhibitory agents of this pathway [40]. However, it has remained unclear whether UDCA affects ERK1/2 activation. The dysregulated activation of ERK1/2 expression results in malignant transformation, and a high level of phosphorylated ERK1/2 (pERK1/2, activated ERK1/2) has been found to be cellular survival signaling in a variety of human cancerous tissues such as melanoma and breast cancers [41,42]. That UDCA suppressed the phosphorylation of ERK1/2 in HCT116 cells (Figure 4) suggests that UDCA inhibits cancerous HCT116 cell proliferation through inhibiting cellular ERK1/2 survival signaling.

Similarly, as a nuclear protein and downstream target of the ERK1/2 pathway, c-Myc is a primary regulator of cell proliferation, cellular transformation, and apoptosis [43]. In addition, c-Myc acts as a key regulatory cellular protein in cell cycle progression, and its downregulation results in cell cycle arrest [5,43]. Thus, our data showing that UDCA drastically reduced the c-Myc protein level in HCT116 cells (Figure 4) may be a major causal factor because c-Myc protein is essential to regulating the protein levels of both cyclins and cyclin-dependent kinases for the control of progression from G0/G1-phase cells to S-phase cells [43,44].

The proto-oncogene c-Myc tightly controls cell proliferation in normal cells, but it is overexpressed and dysregulated in most human cancers [45]. In contrast, p21 is a putative tumor suppressor and a cyclin-dependent kinase inhibitor 1A (CDKN1A) [46]. Our data revealed a prominent dose-dependent increase in p21 protein levels induced by UDCA treatment (Figure 4). This aligns with the fact that p21 is critical in controlling cell cycle progression, especially the G1-phase cell cycle checkpoint, and is intimately associated with cellular events such as cell proliferation and tumorigenesis [47,48]. For this reason, a UDCA-induced increase in p21 protein may play a primary role in cancer cell cycle arrest in HCT116 cells (Figure 2 and Figure 4, Table S1). Moreover, consistent with the fact that c-Myc is upstream of and transcriptionally represses p21 [49,50], our data also demonstrated that UDCA increased the p21 protein level while downregulating c-Myc in HCT116 cells (Figure 4).

Although heterochromatic regions are located close to the nuclear periphery, transcriptionally active euchromatic genomic regions of c-Myc are observed in the inner portions of cell nuclei [31]. In view of UDCA’s high inhibitory potential, we then examined its impact on the subcellular localization of c-Myc protein. In addition to the UDCA inhibition of c-Myc protein levels, few condensed c-Myc protein patches were found in the inner parts of the nuclei in the HCT116 cells after treatment with 0.3 or 0.4 mM UDCA in comparison to the untreated cells (Figure 5). This observation indicates that UDCA reduces c-Myc protein levels and transcription in HCT116 cells. Because c-Myc may promote cancer cell proliferation and anti-apoptosis by suppressing p21 at the transcriptional and post-transcriptional levels [49,50,51], these findings suggest that UDCA reduces c-Myc protein levels and subsequently increases p21 expression, leading to G1 cell cycle arrest in HCT116 cells. Altogether, UDCA plausibly reduces cancerous HCT116 cell proliferation through the suppression of the ERK1/2/c-Myc/p21 signaling pathway.

The putative tumor suppressor protein p21 is a critical regulator with multiple functions in cell cycle, apoptosis, and gene transcription [47]. The upregulation of the cellular p21 protein level can lead to both cell cycle arrest and apoptosis [47]. In the context of apoptosis, the key function of PARP-1, a nuclear protein, is to prevent apoptotic processes through genome maintenance. PARP-1 is a major substrate cleaved by caspase 3 and 7, and this cleavage is a hallmark of apoptosis [52,53]. PARP-1 fragments (apoptotic signatures) were increased in HCT116 cells treated with UDCA (Figure 3 and Figure 4). These data suggest that, in addition to cell cycle arrest, UDCA also inhibits cancerous HCT116 cell proliferation through caspase-mediated apoptosis.

Further characterization of UDCA-related apoptotic genes showed the essential role of caspases in apoptotic processes in which UDCA altered the expression of 18 apoptotic genes (Figure 6), 10 of which were pro-apoptotic (Figure 7). They were critically involved in the TNF, NFκB, death receptor BCL-2, p53, and IAP pathways (Figure 7). The protein–protein interaction analysis indicated that there were two interaction clusters: (1) the TNF, NFκB, and death receptor pathways (red cluster); and (2) the BCL-2, p53, and IAP pathways (green cluster) (Figure 7). The protein interaction was more pronounced within the same cluster than between proteins in different clusters (Figure 7). These data suggest that UDCA-induced colon cancer cell apoptosis is due to collective effects in which the potential of pro-apoptotic proteins is greater than that of anti-apoptotic proteins. While the above results are interesting, there are limitations in the present study, in which we only focused on cancerous HCT116 colon cells and noncancerous NCM460 colon cells. There are various colon cancer cell lines derived from cancerous tissues at different stages and differentiation levels. Thus, to generalize UDCA’s inhibitory potential against colon cancer cells, future studies involving many more carefully selected colon cancer cell lines are warranted.

To connect the above mechanistic findings with human clinical data, we also explored c-Myc and p21 protein levels in the ERK1/2-c-Myc-p21 signaling pathway. According to the gene expression analysis profiles of c-Myc and p21 in a cohort of cancer patients, including patients with the top five cancer types in the U.S., the c-Myc mRNA levels in the tumor tissues were increased to the highest level in colon cancer among these major cancer types while accompanying a decrease in p21 mRNA in comparison to normal control tissues (Figure 8). These data suggest that c-Myc acts as a proto-oncogene but p21 functions as a tumor suppressor in tumor tissues of the colon [43,46]. The clinical data on colon cancer (Figure 8), showing that UDCA greatly reduced the protein levels of c-Myc but increased them in p21 (Figure 4 and Figure 5), strongly suggests that UDCA may exert its anticancer potential, at least in part, via the ERK1/2/c-Myc/p21 signaling pathway in vivo.

## 5. Conclusions

**Figure 9:**
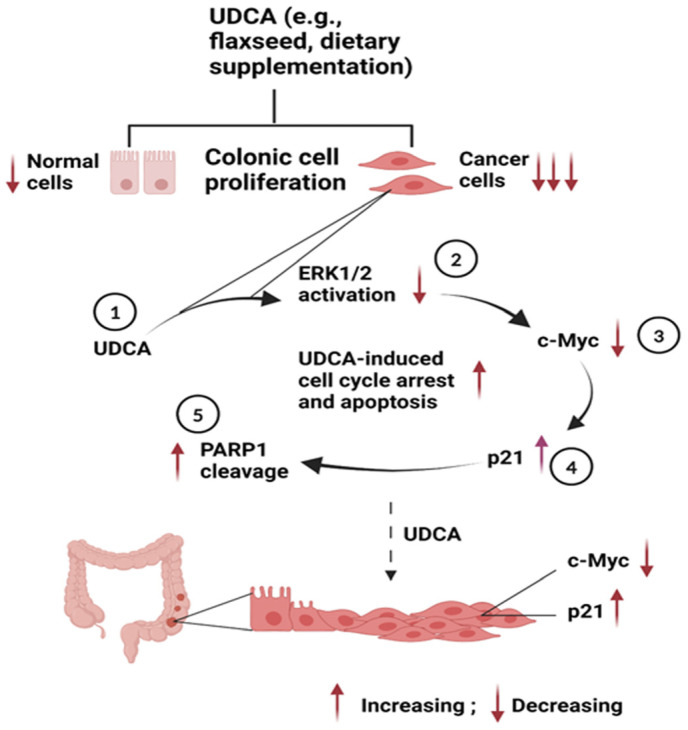
Proposed underlying molecular mechanisms (step 1 to 5) by which UDCA inhibits cancerous HCT116 colon cells to a higher degree than noncancerous NCM460 colon cells.

Collectively, UDCA preferentially inhibits cell proliferation in cancerous HCT116 colon cells with a limited impact on noncancerous NCM460 colon cells. With these cell models, the inhibitory cell proliferation plausibly occurs through cell cycle arrest and apoptosis via the ERK1/2/c-Myc/p21 pathways and the modification of apoptotic gene expression. These mechanistic data provide a proof of concept that may account for UDCA’s anticancer potential in the colon (Figure 9).

## Figures and Tables

**Table 1:** Effect of UDCA on cell cycle progression in HCT116 and NCM460 cells.

UDCA (mM)	0	0.1	0.2	0.3	0.4
Treatment time	24 h				
HCT116 G1%	39.5 ± 3.2 ^c^	38.9 ± 1.6 ^c^	43.4 ± 2.4 ^bc^	48.8 ± 2.3 ^b^	58.3 ± 2.9 ^a^
HCT116 S%	27.3 ± 2.0 ^a^	27.1 ± 1.5 ^a^	26.0 ± 1.6 ^ab^	23.0 ± 1.3 ^b^	19.5 ± 1.6 ^c^
HCT116 G2%	33.2 ± 1.8 ^a^	34.0 ± 0.9 ^a^	30.6 ± 1.4 ^ab^	28.2 ± 2.8 ^b^	22.2 ± 2.1 ^c^
NCM460 G1%	43.9 ± 2.2 ^c^	43.5 ± 2.4 ^c^	46.5 ± 3.5 ^bc^	49.5 ± 2.5 ^b^	55.1 ± 1.1 ^a^
NCM460 S%	28.7 ± 2.1 ^a^	30.4 ± 3.1 ^a^	28.6 ± 4.1 ^a^	27.8 ± 0.9 ^a^	25.2 ± 2.0 ^a^
NCM460 G2%	27.2 ± 0.9 ^a^	26.1 ± 3.5 ^ab^	24.9 ± 1.6 ^ab^	22.8 ± 1.6 ^bc^	19.7 ± 1.3 ^c^
Treatment time	48 h				
HCT116 G1%	37.4 ± 1.2 ^d^	39.6 ± 1.8 ^cd^	43.6 ± 2.1 ^c^	48.7 ± 2.6 ^b^	55.8 ± 2.8 ^a^
HCT116 S%	26.8 ± 1.8 ^a^	25.2 ± 1.2 ^a^	24.8 ± 1.0 ^a^	21.0 ± 1.6 ^b^	18.5 ± 2.0 ^b^
HCT116 G2%	35.8 ± 2.1 ^a^	35.2 ± 0.6 ^a^	31.6 ± 1.7 ^b^	30.3 ± 1.7 ^b^	25.5 ± 1.6 ^c^
NCM460 G1%	41.6 ± 1.2 ^c^	41.7 ± 1.6 ^c^	45.4 ± 1.4 ^b^	48.6 ± 1.8 ^a^	51.8 ± 1.1 ^a^
NCM460 S%	29.0 ± 1.7 ^a^	30.0 ± 2.5 ^a^	28.6 ± 2.4 ^a^	26.9 ± 1.9 ^a^	26.6 ± 2.0 ^a^
NCM460 G2%	29.4 ± 1.5 ^a^	28.3 ± 1.7 ^ab^	26.0 ± 2.1 ^ab^	24.5 ± 1.9 ^bc^	21.5 ± 2.2 ^c^

Values are means ± SDs, n = 4 independent experiments. In a given row, when two values share at least one letter, then the difference between them is not statistically significant. However, when two values do not have a letter in common, then the difference between them is statistically significant; *p* < 0.05.

**Table 2:** Apoptotic gene expression in HCT116 cells treated with UDCA for 48 h.

Gene	Cluster	Control, [0 mM]	UDCA, [0.4 mM]
*Bad*	Green	1.00 ± 0.09	0.27 ± 0.14 *
*BID*	Green	1.00 ± 0.15	0.28 ± 0.12 *
*BIRC3*	Green	1.00 ± 0.05	4.56 ± 2.34 *
*BIRC5*	Green	1.00 ± 0.10	0.26 ± 0.14 **
*CASP2*	Green	1.00 ± 0.04	0.57 ± 0.12 **
*CASP4*	Green	1.00 ± 0.03	2.02 ± 0.39 **
*DAPK1*	Green	1.00 ± 0.03	0.44 ± 0.04 **
*RELA*	Green	1.00 ± 0.04	0.41 ± 0.14 *
*PIDD1*	Green	1.00 ± 0.03	0.38 ± 0.09 **
*CASP8AP2*	Red	1.00 ± 0.05	0.40 ± 0.19 **
*CFLAR*	Red	1.00 ± 0.04	1.77 ± 0.63 *
*DEDD*	Red	1.00 ± 0.04	0.59 ± 0.10 **
*DEDD2*	Red	1.00 ± 0.05	0.39 ± 0.15 **
*FAS*	Red	1.00 ± 0.05	2.64 ± 1.24 *
*NFKBIB*	Red	1.00 ± 0.11	0.25 ± 0.08 **
*NFKBID*	Red	1.00 ± 0.01	0.77 ± 0.10 *
*PEA15*	Red	1.00 ± 0.14	0.15 ± 0.07 **
*TNFRSF25*	Red	1.00 ± 0.02	1.66 ± 0.27 **

* Values are means ± SDs, from independent experiments (n = 4). * *p* < 0.05 and ** *p* < 0.01, relative to untreated HCT116 cells after FDR-corrected Student *t*-tests.

## Data Availability

The data in this study can be obtained upon request from the corresponding author.

## Supplementary Materials

The following supporting information can be downloaded at: https://www.mdpi.com/article/10.3390/nu17061072/s1, Table S1: Effect of UDCA on the cell cycle progression in HCT116 and NCM460 cells.

## Abbreviations

c-Myc, cellular myelocytomatosis oncogene; ERK1/2, extracellular-regulated kinase 1/2; PI, propidium iodide; UDCA, ursodeoxycholic acid; FBS, fetal bovine serum; PBC, primary biliary cirrhosis; PARP, Poly (ADP-ribose) Polymerase; p21, cyclin-dependent kinase inhibitor 1A (CDKN1A); SC, squamous cell carcinoma.
